# The Impact of a Naturally Occurring Retirement Community Supportive Services Program on Older Adult Participants’ Social Networks: Semistructured Interview Study

**DOI:** 10.2196/37617

**Published:** 2022-11-21

**Authors:** Christine Marie Mills, Simone Parniak, Carri Hand, Colleen McGrath, Debbie Laliberte Rudman, Cassandra Chislett, Mariah Giberson, Lauren White, Vincent DePaul, Catherine Donnelly

**Affiliations:** 1 School of Rehabilitation Therapy Faculty of Health Sciences Queen's University Kingston, ON Canada; 2 Health Services and Policy Research Institute Queen's University Kingston, ON Canada; 3 School of Occupational Therapy Faculty of Health Sciences Western University London, ON Canada

**Keywords:** aging in place, naturally occurring retirement communities, social networks, social networking, social capital, aged

## Abstract

**Background:**

Most older adults want to age in place, in their homes and communities. However, this can be challenging for many, frequently owing to lack of supports that allow for aging in place. Naturally occurring retirement community supportive services programs (NORC-SSPs) offer an approach to help older adults age in place. Although qualitative studies have examined the experiences of NORC-SSP participants, little is known about how participation in NORC-SSP programming affects participants’ social networks.

**Objective:**

This study aimed to explore the experiences of 13 NORC-SSP residents who participated in Oasis Senior Supportive Living (Oasis) and how participating in NORC-SSP programming, specifically based on the Oasis model, influenced their social networks.

**Methods:**

Participants were recruited, using convenience sampling, from 4 naturally occurring retirement communities (NORCs) in Ontario, Canada. All participants (13/13, 100%) had participated in Oasis programming. Semistructured qualitative interviews were conducted with participants. Social network theory informed the interview guide and thematic analysis.

**Results:**

In total, 13 participants (n=12, 92% women and n=1, 8% men) were interviewed. These participants were from 4 different NORCs where Oasis had been implemented, comprising 2 midrise apartment buildings, 1 low-rise apartment building, and 1 mobile home community. Overall, 3 main themes were identified from the interviews with Oasis participants: expansion and deepening of social networks, Oasis activities (something to do, someone to do it with), and self-reported impact of Oasis on mental health and well-being (feeling and coping with life better). Participants noted that Oasis provided them with opportunities to meet new people and broaden their social networks, both within and outside their NORCs. They also indicated that Oasis provided them with meaningful ways to spend their time, including opportunities to socialize and try new activities. Participants stated that participating in Oasis helped to alleviate loneliness and improved their quality of life. They noted that Oasis provided them with a reason to get up in the morning. However, the experiences described by participants may not be reflective of all Oasis members. Those who had positive experiences may have been more likely to agree to be interviewed.

**Conclusions:**

On the basis of the participants’ interviews, Oasis is an effective aging-in-place model that has been successfully implemented in low-rise apartment buildings, midrise apartment buildings, and mobile home communities. Participating in Oasis allowed participants to expand their social networks and improve their mental health and well-being. Therefore, NORCs may offer an ideal opportunity to build strong communities that provide deep, meaningful social connections that expand social networks. NORC-SSPs, such as Oasis, can support healthy aging and allow older adults to age in place.

## Introduction

### Background

Older adults are the fastest-growing demographic group in Canada [1], and most of them want to age in place within their communities [2]. Aging in place means that an older adult’s health, access to services, and social support interact in ways that enable living safely and autonomously in their homes or their communities for as long as they desire [3]. Aging in place, although desired by most older adults, is challenging for many. Approximately one-fourth of older adults report feeling isolated [4,5], and between 20% and 34% indicate that they are lonely [6]. Loneliness and social isolation are associated with increased risk of mortality and morbidity**,** representing as strong a risk factor for premature death as smoking [7].

The World Health Organization defines healthy aging as “the process of developing and maintaining the functional ability that enables well-being in older age” [8]. Building and maintaining relationships are critical for health and well-being and enable older adults to continue to live and participate in their communities. Naturally occurring retirement communities (NORCs), unplanned communities (eg, apartment buildings) with a high proportion of older residents [9], are ideally positioned to support aging in place by integrating programs designed to build social connections and support healthy aging. By their very nature, NORCs are naturally existing high-density areas of older adults, which makes them a natural fit for older adult–focused programs and services, otherwise known as the creation of a NORC supportive services program (NORC-SSP).

A way in which NORCs support aging in place is through these NORC-SSPs. A NORC-SSP “is a community-level intervention in which older adults, building owners and managers, service providers, funders, and other community partners create a network of services and volunteer opportunities to promote aging in place among older adults who live in *naturally occurring retirement communities*, housing developments and residential areas not planned for older adults but in which large numbers of older adults reside” [10]. Thus, the goals of NORC-SSPs are to help older adults to age in place, in their communities, as most of them wish to do [2].

Oasis Senior Supportive Living (Oasis), is an example of a NORC-SSP. Oasis is an innovative aging-in-place model that integrates health and supportive services for older adults in a NORC in partnership with older adults, building owners, researchers, and community partners. The Oasis model was codeveloped with the Kingston Council on Aging in 2011 by a group of older adults living in a Kingston, Ontario apartment building, to address the problem of social isolation and its associated health risks among its residents. Since 2019, Oasis programs have opened in 7 additional NORCs across Ontario, each providing a supportive, socially connected, living environment. Oasis empowers older adults to identify their needs and determine the services and activities that best meet these needs [11]. An on-site Oasis coordinator supports members to implement desired activities and services, including member-led activities such as craft circles, line dancing, potluck meals, or engaging community service providers to provide specific programming including exercise classes in communal meeting spaces. Oasis is novel in its emphasis on improving health through community development, strong social networks, and self-determination in older adult members.

NORC-SSPs were created with the intention of bringing programing to where older adults live to strengthen social connections and promote engagement in activities, with the ultimate goal of supporting older adults to age within their communities. Social network theory posits that social structures play an important role in determining individual behaviors [12] and offers a lens to delve deep into how social networks affect mental health and well-being within NORC-SSPs. Grounded in social network theory, Berkman et al [12] offer a conceptual framework that explores the influence of social networks (at the mesolevel) on individual behaviors (at the microlevel). These social networks and individual behaviors are seen to influence health outcomes, including mental health and well-being [12].

A recent scoping review of NORC literature found that much of the research seeks to define NORCs and describe activities and services meant to build strong social relationships [13]. So far, there has been less focus on the effect of NORCs and NORC-SSPs on social networks. Studies have found that participants living in NORC-SSPs have a greater sense of community [14-20], experience enhanced social support, and report reduced social isolation [11,15,17-19,21-25]. A handful of qualitative studies have been conducted with participants living in NORCs; one study sought to understand the factors that influence member participation in NORCs [26] and another study sought to gain perspectives of members living in a NORC as part of an evaluation to understand the implementation of programming in a NORC [27]. So far, no studies have sought to understand how and in what ways a NORC or NORC-SSP influences social networks and how these social networks in turn affect health and well-being.

### Objectives

The purpose of this study was to (1) understand older adults’ experiences of participating in Oasis; (2) explore how participation in Oasis influenced their social networks at the mesolevel and microlevel, using the social network theory by Berkman et al [12]; and (3) understand how these social networks influenced participants’ health and well-being.

## Methods

### Approach

This study used a qualitative descriptive approach to examine the experiences of participants and their social networks (as described by Berkman et al [12]) within a NORC-SSP model, Oasis [28,29]. Qualitative description is grounded in the philosophical tenets of naturalistic inquiry [30] and is an approach to qualitative research that offers a summary and understanding of experiences and includes contextual factors that shape these experiences [31]. A qualitative descriptive study is guided by a theoretical framework, and the social network theory by Berkman et al [12] guided this study, including the development of the interview guide, identification of the variables and relationships to be explored, and analysis. For this study, we were interested in Oasis participants’ experiences, how their experiences affected their social networks, contexts in which the experiences occurred, and how participation in Oasis influenced their health and well-being, thus making qualitative description appropriate for this study and making the social network theory by Berkman et al [12] an appropriate theoretical framework.

### Participants

Participants included a sample of older adults who participated in the Oasis program at 4 different locations in Ontario, Canada. The NORC contexts in which the Oasis program was implemented included 2 midrise apartment buildings, 1 low-rise apartment building, and 1 mobile home community. Recruitment continued until data saturation had occurred, that is, until interviewers repeatedly heard statements similar to those heard in previous interviews [32]. A total of 13 participants (n=12, 92% women and n=1, 8% men) representing the 4 Oasis communities were interviewed. The Oasis program had been implemented approximately 1 year before the interviews, thus ensuring that participants had experience with and engagement in the program.

### Semistructured Interview Guide

Consistent with a qualitative descriptive approach that recommends theory-informed data collection, the social network theory by Berkman et al [12] supported the development of the interview guide (Multimedia Appendix 1). The interview guide sought to explore the macroenvironment that may have influenced social networks and included questions about sociostructural factors (such as building dynamics). Questions also examined the mesoenvironment and explored social networks, including who was in their social network, influence of Oasis on social networks, and psychosocial mechanisms of these networks including the types of supports they received, and Oasis programs they participated in. Finally, the interview guide included questions about potential health pathways, including questions about how Oasis affected daily routines and overall health and well-being.

### Ethics Approval

Ethics approval and consent were obtained from the Queen’s University Health Sciences and Affiliated Teaching Hospitals Research Ethics Board in Kingston, Ontario (REH-722-18).

### Procedure

Participants were recruited by inviting older adults who were Oasis members to participate in the study. The study was conducted at the beginning of the COVID-19 pandemic, and owing to physical distancing protocols, all interviews were conducted via telephone during March 2020 and April 2020. Consent was obtained verbally. The interviews were audio-recorded and, subsequently, transcribed verbatim and analyzed.

### Analysis

The aims of the analysis were to explore and understand experiences of the Oasis participants; therefore, thematic analysis was chosen as the analytic strategy. Thematic analysis, as described by Braun and Clarke [33], is a qualitative descriptive approach that is used to identify, analyze, and report patterns, called themes, within data. It is useful for “analyzing narrative materials of life stories” [34].

NVivo (version 12; QSR International) was used to conduct the thematic analysis of the transcripts. Social network theory [12] provided the conceptual framework for the development of the initial themes. Overall, 6 authors (CC, CD, MG, C Mills, SP, and LW) independently read one of the transcripts to develop a list of preliminary codes based on the conceptual framework. Then, this transcript and the codes each author had developed and applied were reviewed line by line as a group, and a preliminary code book was developed. Then, the 6 authors collated the codes into potential themes by grouping similar codes together. Then, 5 authors (CC, CD, MG, C Mills, and LW) were each assigned several transcripts to code; a second member of this coding team then reviewed how those transcripts had been coded. The codes were discussed until consensus was reached. Then, the same 6 authors met again as a group, reviewed one of the transcripts, and further refined the codes and themes until consensus was reached, and a master list of final codes and themes was developed. Then, each of these 6 authors returned to the transcripts they had been assigned to apply the master list to segments of text and to highlight quotes that provided strong illustrations of the themes. Then, 2 authors (C Mills and SP) reviewed the chosen quotes to find exemplars for each theme.

## Results

### Sample Description

In total, 13 participants from 4 different Oasis communities were interviewed. Of these 13 participants, 12 (92%) identified as women and 1 (8%) identified as a man. The represented Oasis communities consisted of 2 midrise apartment buildings, 1 low-rise apartment building, and 1 mobile home community. Overall, 3 broad themes were identified corresponding to the 3 levels in the theory by Berkman et al [12].

### Social Networks (Mesolevel)—Expansion and Deepening of Oasis Member Social Networks  

#### Participants’ Experiences

At the mesolevel, it was clear from the interviews that Oasis provided older adults the opportunity to expand their social networks. A participant shared the following: 

[Oasis] gave us...an opportunity to meet people and live beyond our own walls. Expand us. Which was really a positive thing. Because you can always keep yourself busy at home but then you have no social network, you have no friends, you have no one to talk to. And you get small when you do that.

Participant 8

Another participant (participant 12) stated, “It just makes you feel more like you’re more welcome to the building.”

Participants noted that Oasis provided them with opportunities to meet new people within their NORC and helped them to broaden their social network. A participant (participant 8) stated, “I was pretty social before this started but just in here there’s a lot more people I know...that’s just made my social group larger.”

Another participant (participant 6) shared, “I’ve lived here for four years, and I met more people when Oasis was going on than I had in the whole four years before that.”

Expanding their social networks meant that participants had more people they could call on for support. A participant shared the following:

It’s [social network] gotten a bit bigger. Before I only socialized with maybe 2, 3 people and since, Oasis has opened up a whole new group of friends that I can rely on and they can rely on me.

Participant 13

#### New Friendships

In addition to the increase in social network size, participants spoke of new friendships that they had formed with other Oasis participants. Through the social components of Oasis, participants were able to connect with other older adults and form meaningful relationships that extended beyond the Oasis setting. A person shared the following:

I just gained so much from Oasis. I gained friends, I gained friendships, I gained companionship. I gained a lot of empathy because there was a lot of people who were a lot worse off than I am, that I was able to empathize with and show compassion for.

Participant 5

Another participant stated the following:

We have some very tight friends we’ve made because of this. And that’s expanded our community.

Participant 8

This participant later added the following:

[Oasis] kept us all getting to know each other even better and better. So, we’ve made some wonderful friends here. Wonderful.

Participant 8

#### Deepening of Previous Friendships With Neighbors

Oasis also strengthened participants’ previous social networks. Participants emphasized that Oasis allowed them to deepen friendships that they had previously; this resulted in participants feeling more closely connected to residents of their NORC. A participant shared the following:

I think it strengthened it [my social network] here in the building and I saw those people that I mentioned more rather than less. Having a meal together once a week. Going to coffee hour...going to craft mornings occasionally.

Participant 3

Another participant (participant 11) stated, “I developed deeper friendships with the people in the building.” This participant later added, “It [Oasis] just made me closer to people and makes better connections.”

#### Comfort in Knowing That Friends Are Nearby and Available to Provide Support If Needed

It appeared that participants appreciated the sense of comfort that came with having close connections and friendships with other residents of their NORC owing to participating in Oasis. Participants often spoke about how nice it was to know that they had people in their social network whom they could rely on when they needed various kinds of support. A participant (participant 3) stated, “I always had a support system but it’s larger now.”

Another participant shared the following:

It’s still just comforting, the only way I can describe it is just very comforting to know I can pick up the phone or I can take my walking poles and walk down to one of my many friends and say, “I need a coffee and a hug” and that’s exactly what we get.

Participant 4

Oasis participants began to feel similar to family. A participant shared the following:

There’s a gentleman here, a lovely gentleman, he’ll be [over 85], I believe. He had lived here for, I believe, 17 or 20 years and basically knew nobody. And Oasis is now what he refers to as his family.

Participant 7

#### Positive Relationship With Oasis Coordinator

Oasis expanded older adults’ social networks, not only through interacting with other Oasis participants, but also through their relationship with the Oasis coordinator. Participants expressed positive feelings toward the Oasis coordinator and the significant role that they played. The support the coordinator provided to Oasis participants was appreciated. Throughout the interviews, it was clear that Oasis participants value the Oasis coordinator as part of their social network. A participant shared the following:

[The coordinator] is a lovely person, she did the exercises, and she was lots of fun...She calls me about once a week to see how I’m doing and that’s wonderful to think that somebody cares and says that if you need anything or want to talk to somebody just give me a ring.

Participant 2

Another participant echoed this view:

If they did have any problems they’d talk to [the coordinator] about things that was private between her and them, but they did know that if they needed help, they could go down and talk to her.

Participant 1

Participants appreciated the work the coordinator put into activities: participant 11 stated, “The coordinator was excellent, she arranged all these ideas...[you] could talk to her about anything.”

The coordinator would try to get answers to participants’ questions:

Very helpful and very friendly and very informative...“oh, you want to know about that? I’ll look into that for you, I’ll see where I can get some answers for you.”

Participant 7

This was echoed by another participant:

She was very good, and she was very friendly, and she’s very organized and helping everybody in lots of ways and helping get us information...If there was something we wondered about and she didn’t know, she would look into it for us and get back to us. She was just all around doing a wonderful job.

Participant 8

The coordinator was a key part Oasis’ success:

What I would take away from this program is that it proved to me how valuable it is for a naturally occurring retirement community to be supported in this way, with an on-site coordinator.

Participant 3

#### Community Partnerships

Oasis also provided participants with the opportunity to expand their social networks even further, to the broad community. Participants valued the guest speakers and community partners who were brought into Oasis. Oasis helped to connect older adults to their community by increasing their knowledge of services that are available. A participant said the following:

There was a speaker series and so we had, people from organizations such as Diabetes and the Hearing Society and, Canadian National Institute for the Blind...We had the Law Society from Queens, the Elder Law Clinic, the student who ran that came. Some of us got our wills redone who hadn’t redone them since we’d lost a spouse.

Participant 3

This participant later added, “The Oasis program gave me access to guest speakers and organizations in an easy way.”

Oasis participants appreciated many of the topics discussed:

They had a speaker in once a month...they had so many different ones...I found that very interesting, and we were learning at the same time.

Participant 2

This was echoed by another participant:

We have a lot of speakers come in about nutrition, about falling, balance, things like that, about pharmacists...They were really good, really informative.

Participant 11

Oasis provided participants with the opportunity to expand and deepen their social networks. Participants indicated that other Oasis participants and the Oasis program coordinator provided them with support. Participating in Oasis also expanded their knowledge of community programs and services.

### Individual Behaviors (Microlevel)—Oasis Activities: Something To Do, Someone To Do It With

#### Participants’ Experiences

As participants described their experiences with Oasis, it was evident that programming provided participants an opportunity to engage in meaningful activities with other people. Participants noted that they enjoyed doing fun activities in a warm social atmosphere. A participant shared the following:

You know, having coffee and basically laughing, which was I think the primary wonderful aspect of this whole thing. The laughter that was ensuing every time we went down there [to the common room], and the camaraderie was just absolutely heartwarming.

Participant 7

#### Socialization

When asked to describe what they liked about the programming, most participants (12/13, 92%) described both activities and socialization opportunities the activities provided. For example, a participant (participant 13) shared, “I really looked forward to seeing the people, talking with the people and then doing the exercises.”

Similarly, another participant (participant 2) stated, “They had books with puzzles in and everything and we had coffee and we just socialized and talked, and laughed, and had fun.”

Talking and laughing were noted repeatedly by Oasis participants as core elements of the regularly scheduled programming. A participant stated the following:

There was socialization. Doing things that we had fun doing. We’d go down and play Bingo and we’d kill ourselves laughing about something stupid.

Participant 1

#### New Activities

In addition to fun, Oasis programming provided participants with a wide variety of new activities to engage in. A participant described Oasis activities as “new adventures” and was appreciative that the coordinator was “always bringing in different things” (participant 8). Another participant (participant 12) stated, “I’ve learned about some things that I never knew before.”

Types of activities differed by site, but included various crafts, coloring, card and board games, line dancing, movie afternoons, painting, bingo, and social coffee hours, among others. Many participants (7/13, 54%) tried new activities, including different types of crafts. A participant shared how much she enjoyed the new sewing class:

I love the sewing...that’s new on my plate. Never sewed before!

Participant 10

Participants noted that the variety provided by Oasis allowed them to choose activities that suited their interests. Another participant (participant 4) explained, “When I looked at the program, there was something for everyone.”

#### Meaningful Ways to Spend Time

Participants noted that Oasis activities provided them with a meaningful way to fill otherwise vacant time. They expressed the value of having activities to break up long days and get them out of their apartments. A participant (participant 7) shared, “Mondays I would usually just go down for a coffee for an hour or so, just enough to feel like you weren’t alone stuck in your house.”

As activities were organized and run by a combination of the coordinator, community partners, volunteers, and other Oasis participants, the calendars filled up quickly. A participant (participant 8) exclaimed, “...This is getting so busy that we have to pick and choose!”

When asked if she enjoyed being very busy with Oasis activities, a participant (participant 4) remarked, “Well yes, a lot of us did because some days, you know, the days are long.”

Participants also indicated that they liked the routine and looked forward to programming days. A participant (participant 2) shared, “I was always glad when Tuesday, Wednesday, and Thursday came because there were different things going on each day.”

Of note is the emphasis participants placed on the value of simply knowing that there were activities to look forward to in the future. The positive sense of anticipation these future activities provided was a mood booster when they were at home with nothing to do. A participant explained the following:

It’s something to look forward to and I think that’s a key. That when you’re a senior that you’re just not locked into your building, that there are things that you look forward to.

Participant 4

Both future anticipation and fun had in the moment were motivating factors.

Oasis provided the participants with something to do and someone to do it with, which directly aligns with the program’s aim to promote healthy aging within NORCs. 

### Health Outcomes—Impact of Oasis on Self-reported Mental Health and Well-being: Feeling and Coping With Life Better

#### Participants’ Experiences

Oasis participants reported experiencing increased overall well-being. In addition, many participants (7/13, 54%) noted that having fun activities to do and look forward to improve their self-reported mental health. A participant shared the following: 

Well of course I feel so much better because I find that I’m able to cope with life better than I used to...I guess if it hadn’t been for Oasis, I don’t know what I’d be doing or what I’d be like now. I would have probably jumped over this balcony or something...Joking [laughing].

Participant 9

#### Well-being Before Oasis

When asked about their self-perceptions of well-being before the existence of Oasis in their community, a participant (participant 4) explained, “On a scale of 1 to 10, there were days when I was just severely depressed and isolated.”

This participant later expanded on this feeling of depression by adding the following:

We’ve all had those days where [we think] why should I bother getting dressed, and brush my teeth, and have a shower? I’m not going see anybody. There would be weeks go by...where we would never see a soul, or talk to neighbors, or anything.

Participant 4

Repeatedly, participants described their feelings before joining Oasis as “lonely,” “lonesome,” and “isolated.” After Oasis, their mental outlook improved. A participant shared the following:

It gave me something to get up for and something to look forward to everyday. And I didn’t ever have that in my life before, it was a very, very depressing life.

Participant 5

#### Alleviating Loneliness

There were participants who, when referencing their mental health struggles, discussed how the positive atmosphere of Oasis affected their feelings of loneliness and gave them an opportunity to re-engage. A participant shared the following: 

I used to sit here. I was very lonely. Then once Oasis started it was a different story. It really brought me out and got me going again.

Participant 9

This participant later added, “I don’t feel isolated anymore, and I don’t feel lonesome.”

This was echoed by several participants (11/13, 85%):

You don’t feel alone. You might live alone, but you don’t feel alone.

Participant 4

You feel like you’re not all alone in the world.

Participant 3

I didn’t feel so alone. I didn’t feel so isolated.

Participant 5

#### A Reason to Get Up

Oasis clearly provides more than social support; it is a program that individuals heavily rely upon to help them cope with life and a variety of aging-related challenges. Participants stated that Oasis activities provided a reason to get up, get dressed, and proceed with the day when they otherwise may not have. A participant shared the following: 

That’s where programs like Oasis make all the difference. They get people active...human beings have to have a reason to get up in the morning.

Participant 8

The same participant later shared the following:

People need that reason. If you don’t have a reason to get out of bed and get moving and you don’t have anyone to share what you’re doing or your thoughts with, it gets pretty depressing and pretty lonesome.

Participant 8

Having “something to get up for” was reiterated by many participants (6/13, 46%). For instance, participant 5 stated, “Like I said it literally gave me a reason to get up.”

Another participant stated the following:

It was something to look forward to, something to get dressed for. I bought better clothes.

Participant 11

Having a reason to get up and get dressed led to increased motivation to engage in other activities. A participant shared the following:

It was great, it was motivational, it was stimulating. It was enough, to say okay, you know, now that I’ve finished there, I’m going go out and do this because I’m already dressed.

Participant 4

Oasis provided participants with a sense of purpose that was beneficial in helping them to maintain their mental health and well-being. When questioned about how Oasis affected their life, a participant shared the following: 

It really gave [me]...a lift, you know, in my life, that I hadn’t felt for a long time...it really gave me a lift.

Participant 11

Another participant stated the following:

It’s changed my everyday life...it’s just nice when you get up in the morning and you have your coffee...you have friends.

Participant 8

When asked how Oasis affected their overall well-being, this participant described the impact by sharing, “I can’t say anything short of a hundred percent.” 

#### Quality of Life

The main purpose of Oasis is to build healthy communities for older adults within NORCs that are supportive, foster independence, and improve overall quality of life, to support aging in place. Oasis participants reported improvement in their mental health, well-being, and overall quality of life. A participant, when asked about the impact Oasis had on her daily routine and her outlook on life, stated the following: 

It has made me feel better, everyday...I think it does make you feel more like living. When you’re alone lots of time you’re thinking about “oh I’m going downhill,” you feel like you’re dying sometimes. And when you’re at Oasis it builds you up, and you feel more lively.

Participant 2

Another participant (participant 3) said, “I have been happy to see the positive impact this program [Oasis] has had.”

Another participant (participant 4) commented, “I think my life is better for it with Oasis then it would have been without it.”

The powerful influence Oasis had on mental health and well-being was experienced by many participants (11/13, 85%). All the participants who were interviewed (13/13, 100%) commented on this important aspect of Oasis, highlighting the positive impact Oasis had on those who choose to participate in the program. 

## Discussion

### Principal Findings

The purpose of this qualitative study was to explore the experiences of older adult participants who are attending the Oasis program at 4 unique NORCs in Ontario, Canada. To the best of our knowledge, this study offers the first exploration of experiences of older adults living in a NORC through the lens of the social network theory by Berkman et al [12], which describes how social networks influence health and well-being. Participants reported that their involvement in Oasis influenced their social networks, personal behaviors, and health. This study also adds to the NORC literature by examining how participation in Oasis, a NORC-SSP, influenced participants’ social networks, both within and beyond their NORCs.

Overall, 3 main themes were identified from the interviews with Oasis participants: expansion and deepening of social networks, Oasis activities (something to do, someone to do it with), and impact of Oasis on mental health and well-being (feeling and coping with life better).

At the mesolevel of the social network theory by Berkman et al [12], Oasis provided participants with an opportunity to expand and deepen their existing social networks. Participants reported that the number of individuals in their social networks increased, they made new friends, and previous friendships deepened. Participating Oasis members were comforted by the fact that friends were nearby and could be counted upon if they needed support. Oasis participants also discussed their positive relationship with the on-site Oasis coordinator. In addition, Oasis participants also spoke about the influence that Oasis had on their ability to make new connections with a variety of community organizations. To the best of our knowledge, this finding of the role of a NORC-SSPs in broadening of social networks beyond the *walls* of the NORC itself to professional services and organizations, has not been identified previously.

At the microlevel [12], participation in Oasis affected behavior. Oasis provided participants with opportunities to socialize and try new activities and meaningful ways to spend their time. Uniquely, Oasis participants reported trying and engaging in new activities.

Regarding the health outcomes of the social network theory by Berkman et al [12], participants reported the positive impact that Oasis had on their mental health and well-being, alleviating loneliness, and improving quality of life. Notably, and unique to this study, Oasis participants reported that Oasis activities provided them with a reason to get up every day and provided something to look forward to.

### Comparison With Previous Studies

The 3 main themes found in this study are similar to those found in studies examining NORCs in the United States. For example, a study of NORCs in Cleveland, Ohio, found that, when asked what they liked best about their NORC, four themes emerged: interaction with neighbors and making friends, activities or services, resource coordinators, and choice or variety of activities or services [17].

At the mesolevel, Oasis participants reported making new friends and deepening existing friendships. Similarly, other studies of NORC-SSPs have also found that residents made friends with other individuals in their building and had more contact with their neighbors [17,19,23,24,35,36]. The social support provided by other Oasis participants is something that has also been experienced in other NORC-SSPs [19,23,36].

A positive relationship with the on-site Oasis coordinator was something mentioned by many Oasis participants (10/13, 77%). The importance of an on-site program coordinator in supporting NORC activities has also been identified in previous studies of structured NORC-SSPs [17,25,35] and, this highlights the importance of incorporating formal supports into NORC-SSPs, including paid staff, when appropriate. In their survey of 191 older adult participants of the Community Options NORC-SSP in Ohio, Anetzberger [17] indicated that participants repeatedly made unprompted references to the positive effect of the *resource coordinator* on their access to activities and resources and in empowering them in decision-making around programming.

At the microlevel, similar to several other NORC-SSPs, Oasis empowered its members, as they had the opportunities to choose what activities they wanted to engage in and felt they needed [10,17,37]. Other NORC studies have also reported that NORC-based activities provided residents with *something to do* [17,22,24,25]. For example, Anetzberger [17] found that NORC residents appreciated the variety of options available to them, including recreational and educational activities and monthly luncheons. In another study by Cohen-Mansfield et al [22], NORC residents engaged in leisure activities offered by a recreation service, such as trips, tours, and educational programs.

Participants reported that participating in Oasis positively influenced their health and well-being. Participating Oasis members reported decrease in isolation, which is something that has also been reported in other NORCs [10,22,23]. The decrease in depression reported by some Oasis participants has also occurred in other NORCs [22].

### Strengths and Limitations

First, the results of this study are reflective of the experiences of Oasis participants, and therefore, may not be applicable to other NORC-SSPs. Second, Oasis participants who agreed to be interviewed may also be different from those who chose not to be interviewed, and this may have influenced the results. It is possible that participants with more positive experiences or those who were more involved in Oasis would be more willing to share their thoughts with interviewers. Third, data were gathered at only 1 point in time. It is possible that participants’ thoughts and experiences could change over time. However, participants reflected on their lives before Oasis and reported how things had changed since Oasis implementation. Fourth, we did not gather any demographic information beyond gender, and it is possible that ethnicity or age may have affected Oasis participants’ experiences. Fifth, of the 13 participants, 12 (92%) identified as women, leading to our inability to explore the presence of gender-specific experiences with the Oasis program. However, as most participants in Oasis identified as women, our results likely reflect the experiences of many Oasis participants.

Despite these limitations, this study has strengths. First, interviews were conducted with participants from 4 different Oasis sites, indicating that these positive experiences occurred in multiple NORCs in different locations with different program coordinators and reflecting different types of communities. Second, the questions asked provided an in-depth look at Oasis participants’ experiences, thus helping to deliver the rich data presented in this paper.

### Future Directions

Future studies could expand on the thematic findings presented in this paper by further examining the contextual features of the various Oasis sites, such as the types of physical spaces available for activities or the demographic composition of the buildings, to deepen the understanding of how such features shape experiences of the program. Future studies will also examine how physical, social, and psychological factors change over time among Oasis participants.

### Conclusions

On the basis of these findings, Oasis is an effective aging-in-place model because it has expanded and deepened participants’ social networks, increased participants’ engagement in activities, and positively affected participants’ mental health and well-being. Oasis provides a model that has potential to be implemented in other NORCs, as it has been successfully implemented in low-rise apartment buildings, midrise apartment buildings, and mobile home communities. Implementing Oasis programs in additional communities and buildings could address some of the challenges of aging in place and improve older adults’ health and well-being.

## Appendix

Multimedia Appendix 1 Semistructured interview guide.

## Abbreviations

NORC: naturally occurring retirement community
NORC-SSP: naturally occurring retirement community supportive services program
Oasis: Oasis Senior Supportive Living

## Data Availability

The data sets generated and analyzed during this study are available from the corresponding author on reasonable request.
