# Supplementary material for: The Impact of a Naturally Occurring Retirement Community Supportive Services Program on Older Adult Participants’ Social Networks: Semistructured Interview Study
Source: JMIR Aging. 2022 Nov 21;5(4):e37617. doi: 10.2196/37617 (PMC9723974; doi:10.2196/37617)
Supplement: Multimedia Appendix 1 [file aging_v5i4e37617_app1.docx]

Appendix 1: Semi-structured interview guide

**Study: Oasis Seniors Supporting Living: A Model of Active Aging-In-Place**

**Introduction**

Thank you for participating in this interview today. Your insights into your experience with Oasis is greatly appreciated.

*As a reminder, the purpose of this interview is to reflect on your experiences participating Oasis, and share how Oasis has, or has not been beneficial to you.*

This interview should take no more than 60 minutes. The data will be analysed to identify themes. Your responses will remain confidential, and you will be identified in reporting by the use of pseudonyms. Your participation in this interview is entirely voluntary. You are free to withdraw your consent to participate at any time before, during or after the interview. If that is the case, we will not use any information you have provided to us. With your permission the interview will be audio recorded. The recording will be used to ensure accuracy in the analysis of the data and will be destroyed once the project has been completed.

- There are no right or wrong answers.
- Do you have any questions before we begin?  (answers).

Are you still agreeable to participating in this interview? OK, let’s get started.

**Introductory question**

I would like to start by reflecting on how you have participated in Oasis.  Please briefly describe how you have been involved with Oasis.

**Socio-structural factors**

I am going to ask you a few questions about your overall building to get a better understand of how the building dynamics might influence Oasis programming and social interactions.

1. How would you describe the atmosphere in your building?  (prompt – for example is your building a ‘friendly building’, a ‘private building where people keep to themselves’)

1. If there are building issues, how and in what ways would people in your building work together?

1. How would you describe the politics within your building?

1. How do these politics influence how people interact in general?
2. How do these politics influence Oasis?

1. How do any building policies or rules influence how you interact with people in your building?

1. How do any building policies or rules influence Oasis?

**Social Networks**

Social networks are made up of people that you interact with on a regular basis – for example they can include friends, family, neighbors, professionals and others that are part of your life. The next set of questions will ask you about your social network in and outside of Oasis.

1. Who is part of your social network?  (you can name as many people as you would like)

1. Has Oasis impacted (changed) your social network?

1. Can you describe the relationships you have with Oasis members?

1. How frequently would you interact with people in your building?
2. How and in what ways has this changed since the start of Oasis?

1. How frequently would you interact with people face-to-face in the community?
2. How and in what ways has this changed since the start of Oasis?

1. How frequently would you interact with Oasis members?
2. What is the nature of these interactions?
3. How close do you feel to Oasis members?

**Psychosocial mechanisms**

We are interested in understanding how you connect and interact with others.  The next set of questions will focus on the types of interactions that you have with others.

1. What supports do you receive from other people and programs – this might include both formal supports from the health care system or community programs (e.g. homecare), or informal supports from family and/or friends including help with shopping, rides, attending doctors’ appointments?
2. Have these supports changed since starting Oasis?
3. If yes, how and in what ways have these supports changed

1. What types of supports do you receive from Oasis members?
2. What has been the impact of these supports?

1. What Oasis programs do you participate in?
2. Why did you choose these programs
3. Prior to Oasis did you participate in other similar community programs?
4. If yes, how has Oasis changed your participation in these community programs?

1. In what ways has Oasis helped you learn about community programs and services?
2. Have you accessed any of these programs/services (only ask is they indicated they have learned about programs/services)?
3. If yes, what programs have you accessed?

**Health Pathways**

We are now going to focus on the impact Oasis has had on your life and the next set of questions will ask about different aspects of your life.

1. How has Oasis impacted your daily routine?
2. Prompts – has Oasis changed your daily routine, are you doing different activities, meeting different people

1. Did you participate in any of the physical activity programming?
2. If yes, how has Oasis impacted on your physical activities?
3. Prompt –have you been doing different physical activities, more physical activities, met more people doing physical activities?

1. Did you participate in any of the food programming?
2. If yes, how has Oasis impacted on your eating and nutrition?
3. Prompt – have you been eating healthier meals

1. Did you participate in any of the social programming?
2. If yes, how has Oasis impacted on your social connections?

1. How has Oasis impacted on feelings of social isolation?

1. How has Oasis impacted on your ability to cope with changes in your life?

1. How has Oasis impacted your outlook on life?

1. How has Oasis impacted on your overall well-being?

We have two final broad questions.

1. How has Oasis impacted your life?

1. If you had one with for Oasis going forward, what would that be?

**Conclusion**

Thank you for participating. This has been a valuable discussion. Your perspective has all been very important to helping understand Oasis and the impact the program has had.
